# The Predilection of Specific Diseases to Affect Different Sections of the Esophagus

**DOI:** 10.3390/life15091444

**Published:** 2025-09-15

**Authors:** Monjur Ahmed

**Affiliations:** Thomas Jefferson University Hospitals, 132 South 10th Street, Philadelphia, PA 19107, USA; monjur.ahmed@jefferson.edu; Tel.: +1-215-955-8900; Fax: +1-215-755-1850

**Keywords:** predilection of esophageal diseases, eosinophilic esophagitis, esophageal stricture, achalasia cardia, gastroesophageal reflux disease, esophageal cancer

## Abstract

Certain esophageal diseases have a predilection for affecting different parts of the esophagus. This predilection is noted in our clinical practice as we investigate various esophageal diseases using multiple imaging and motility studies, as well as endoscopic procedures with biopsies. Clinical presentations vary with the stage and location of the disease. Clinicians can suspect certain diseases when a particular part of the esophagus is involved and then can perform appropriate investigations. Esophageal diseases with section involvement, with their symptomatology, diagnostic tests, and management, have been discussed in this narrative review. This review aims to revisit those diseases with a current update.

## 1. Introduction

The esophagus is a tubular structure with mucosal, submucosal, muscular, and adventitial layers. Most diseases are mucosal epithelial diseases that can be easily detected with endoscopic procedures. However, many subepithelial diseases require further investigations like imaging and endoscopic ultrasound. Motility disorders are investigated by manometric studies. An interesting fact is that the different parts of the esophagus have different disease predilections. The upper esophagus is mainly affected by cricopharyngeal achalasia, esophageal web, Zenker diverticulum, dermatomyositis, and fibrovascular polyp. Traction esophageal diverticulum can affect the mid-esophagus. Lower esophageal diseases include gastroesophageal reflux disease (GERD), Barrett’s esophagus, adenocarcinoma, achalasia cardia, Schatzki’s ring, epiphrenic diverticulum, and epidermoid metaplasia. Eosinophilic esophagitis (EoE) and esophageal stricture can affect any part of the esophagus. Squamous cell cancer and lichen planus predominantly affect the proximal and mid esophagus. Esophageal leiomyoma, gastrointestinal stromal tumor (GIST) and esophageal scleroderma usually affect the lower two-thirds of the esophagus.

As certain esophageal disorders tend to affect specific parts of the esophagus, this review is important in revisiting these conditions. So only these disorders are included in this review.

## 2. Brief Methods

Electronic databases were searched to get help writing this review. These include ***PubMed Central*** (PMC), ***Google Scholar***, ***Google***, ***and UpToDate***.

## 3. Cricopharyngeal Achalasia (CPA)

The upper esophageal sphincter (UES)dv is a two to four cm high-pressure zone consisting of the inferior pharyngeal constrictor/cricopharyngeal muscle. The normal resting pressure of UES is 34 to 104 mm Hg, and the relaxation pressure is less than 12 mm Hg. However, significant variation can be observed during high-resolution manometry (HRM) [1]. Hypertensive UES is defined as a resting pressure of more than 104 mmHg. Hypotensive UES is defined as a resting pressure of less than 34 mmHg. In CPA, the UES does not relax enough during swallowing. Hypertrophy of the cricopharyngeal muscle leads to CPA. CPA can be primary or secondary to oropharyngeal tumor before and after treatment, cerebrovascular accident (CVA), trauma, thyroid myopathy, dermatomyositis, muscular dystrophy, neuritis, and poliomyelitis [2]. CVA is the most frequent cause of CPA.

Symptoms: Patients typically present with oropharyngeal dysphagia. Other manifestations include coughing, gagging and choking while swallowing liquid food due to laryngeal penetration and aspiration.

### 3.1. Diagnosis

1. Barium swallow shows an unduly prominent smooth posterior impression of the cricopharyngeal muscle at the level of C6 level.
2. Video fluoroscopic swallow studies (VFSS) may show a cricopharyngeal bar at the C6 level.
3. Upper endoscopy reveals a spastic UES that needs pressure to allow the scope to advance.
4. High-resolution manometry is the gold standard for diagnosing CPA. It shows not only high pressure of the UES but also incomplete relaxation [3].

### 3.2. Management

The three treatment tools include endoscopic balloon dilation (EBD), Botulinum toxin injection (BTI) (5 IU/each quadrant), and cricopharyngeal myotomy. EBD may give temporary relief, but BTI has been associated with limited success [4]. Cricopharyngeal myotomy is the treatment of choice and can be performed either through a lateral pharyngotomy approach or a cricopharyngeal peroral endoscopic myotomy (CP-POEM) [5,6]. CP-POEM demonstrate initial symptom relief in about 75–85% of cases, but long-term follow-up reveals requires repeat intervention in 12–25% of cases.

## 4. Esophageal Web

This is a thin membrane 2 to 3 mm in size that protrudes into the esophageal lumen. It consists of mucosa and submucosa. Congenital esophageal webs are rare. They are generally circumferential with a central or eccentric orifice and occur in the middle or distal third of the esophagus. Acquired webs are more commonly observed in clinical practice, typically appearing in the cervical esophagus or the post-cricoid area. Common etiologies include iron deficiency anemia, bullous diseases (such as pemphigus, pemphigoid, and epidermolysis bullosa), graft-versus-host disease, and celiac disease [7].

Symptoms: Most patients with an esophageal web remain asymptomatic. Patients present with intermittent dysphagia or painful swallowing or a feeling of food getting stuck in the esophagus when there is significant narrowing of the esophageal lumen.

### Diagnosis

1. Barium Swallow: esophageal web is seen as a thin, shelf-like filling defect along the anterior wall of the upper esophagus or as a radiolucent ring in the upper esophagus.
2. Upper endoscopy: esophageal web is visualized as a thin, smooth membrane that does not span the whole circumference of the esophagus [8].

Management: Endoscopic dilation (either balloon or bougienage) is the treatment of choice. If endoscopic dilation is inadequate, endoscopic electrocauterization can be followed by balloon dilation [9]. Endoscopic dilation is highly effective for initial symptom relief, but recurrence is not uncommon, particularly in patients with multiple or congenital webs, requiring ongoing surveillance. In complex cases, insulated tip needle knife incision has been reported to be effective [10]. A recognized association exists between a cricopharyngeal web secondary to Plummer-Vinson syndrome and squamous cell cancer of the esophagus. Clinicians should be vigilant about this risk and consider monitoring these patients for early signs of esophageal cancer [11].

## 5. Zenker Diverticulum

This is formed at the pharyngo-esophageal junction between the inferior pharyngeal constrictor muscle and cricopharyngeal muscle (Killian triangle or dehiscence) due to the development of high intraluminal pressure because of incoordination or spasm of the cricopharyngeal muscle or poor compliance of the upper esophageal sphincter [12]. It is a pulsion-pseudodiverticulum consisting of mucosal and submucosal layers of the hypopharynx. More than half of patients with Zenker diverticulum are elderly, between the ages of 60 and 80 years, and it is very rarely seen in individuals under the age of 40 [13].

Symptoms: Patients with a small (<2 cm) Zenker diverticulum may remain completely asymptomatic. Patients with a large diverticulum generally present with dysphagia, regurgitation of undigested food, halitosis, and globus sensation in the throat. Sometimes, patients may present with aspiration pneumonia or significant weight loss [14,15].

### Diagnosis

1. Barium Swallow is considered the gold standard in diagnosing Zenker diverticulum. It shows an outpouching of the posterior pharyngeal wall just above the upper esophageal sphincter at the level of C5–C6. The pouch has a narrow neck that allows barium to be trapped during the swallowing process. On a lateral view of the imaging, an air-fluid level can be seen within the pouch [16].
2. Video fluoroscopy is a dynamic study that can image the real-time swallowing process and the Zenker’s diverticulum.
3. Upper endoscopy is practical both for diagnostic and therapeutic purposes. It may allow visualization of the mucosa of the diverticulum, pooling of food inside the diverticulum, and fibrosis around the diverticulum [17].

Management: Symptomatic patients with Zenker diverticulum should be treated endoscopically or surgically. The treatment of choice is flexible endoscopic myotomy. Endoscopically, about 1 cm of myotomy is performed by cutting the septum (cricopharyngeal muscle) between the esophageal lumen and Zenker diverticulum with a specialized knife (hook knife or needle knife). As a result, a common channel is formed between the esophagus and the diverticulum, allowing food to reach the esophagus easily without obstruction. Most patients get relief from dysphagia after this procedure. This procedure is less invasive, tolerated by elderly patients with comorbidities, and requires a short hospital stay [18]. A subset of patients may develop recurrence of symptoms because of incomplete myotomy, and endoscopic treatment can be repeated in those cases. Reported complications from flexible endoscopic myotomy include bleeding, microperforation, mediastinitis, cervical abscess, throat pain, and transient fever with leucocytosis. Other endoscopic treatments include endoscopic stapling, diverticulectomy, and endoscopic carbon dioxide laser diverticulectomy. Endoscopic treatment is suitable for small to medium-sized diverticula (up to 5 cm) in elderly patients with comorbidities who are not good candidates for surgery. In open surgery (transcervical access), diverticulectomy with concurrent cricopharyngeal myotomy is performed. Surgical complications include pharygocutaneous fistula, parapharyngeal abscess, pneumomediastinum, mediastinitis, aspiration pneumonia, significant bleeding, wound infection, transient recurrent laryngeal nerve palsy, microperforation with subcutaneous emphysema, and postoperative fever. Open surgery should be considered for a large diverticulum in young patients who are good surgical candidates [19].

## 6. Dermatomyositis

This can affect the striated muscles of the pharynx and upper esophagus due to inflammatory myopathy. Complement (C5b-9) mediated microangiopathy leads to capillary loss, ischemia, and muscle fiber necrosis [20]. One in three patients with dermatomyositis can develop oropharyngeal dysphagia for both solid and liquid foods [21].

Symptoms: Patients usually present with oropharyngeal dysphagia that can result in a choking sensation, nasal regurgitation, aspiration pneumonia, and significant weight loss.

### Diagnosis

1. Serology: serum creatinine phosphokinase (CPK), aldolase, lactate dehydrogenase (LDH), aspartate aminotransferase (AST), and alanine aminotransferase (ALT) will be elevated. Anti-transcription intermediary factor 1γ (TIF-1γ) antibody can be positive in more than 80% of patients.
2. Video fluoroscopy swallow study: classic findings include pharyngeal pooling and/or nasal regurgitation [22].
3. Manometry reveals decreased upper esophageal sphincter pressure, low amplitude or absent contraction of pharynx and upper esophagus [23].

Management: High-dose corticosteroids are the first-line treatment. If the patient is resistant to corticosteroids, alternative therapies such as azathioprine, methotrexate, cyclophosphamide, or cyclosporin A can be considered [24]. Intravenous immunoglobulins (IVIG) can be used in life-threatening esophageal involvement due to dermatomyositis [25]. Patients also require rehabilitation therapy, dietary modifications, and specific muscle strengthening exercises to enhance their swallowing function. A multidisciplinary approach is needed, involving dietitians and speech therapists or speech-language pathologists.

## 7. Fibrovascular Polyps or Fibroepithelial Polyps

These are rare, benign intraluminal pedunculated tumor-like lesions that arise from the lamina propria or submucosa and can grow to a considerable length inside the esophagus. Approximately 80% of the time, they originate in the upper esophagus, just distal to the cricopharyngeal muscle, and extend caudally due to the propulsive effect of swallowing. Histologically, the polyp is composed of fibrovascular tissue with some lipomatous and myomatous changes, all covered by normal squamous epithelium [26].

Symptoms: Patients may remain asymptomatic if the polyps are small. As the polyp grows bigger, patients generally present with dysphagia, regurgitation, hiccup and weight loss. Sometimes, the polyp may protrude into the mouth cavity and can obstruct the airway, causing fatal asphyxiation [27].

Diagnosis: Imaging studies, such as a barium swallow or CT scan, may reveal a filling defect in the upper esophagus. Upper endoscopy can visualize the mobile polyp in the esophagus [28].

Management: The treatment of choice is complete surgical resection of the polyp via lateral cervical esophagotomy or thoracotomy, as the polyp is highly vascular. Recurrence of the polyp following surgical resection is rare [29]. Endoscopic resection can be performed depending on the location and size of the polyp [30]. A multidisciplinary team (consisting of an interventional endoscopist, a thoracic surgeon, and a laryngologist) approach is required for successful treatment.

## 8. Squamous Cell Carcinoma of the Esophagus

This can occur at any part of the esophagus but is primarily seen in the upper or mid-esophagus. Globally, this is the most common type of esophageal carcinoma. Risk factors for developing esophageal carcinoma include tobacco smoking, alcohol consumption, organ transplantation, diets low in fruits and vegetables and high in processed food, consumption of very hot beverages and foods, people living in highly endemic areas like ‘esophageal cancer belt’ (Northern Iran, Central Asia and North-Central China), iron deficiency anemia with cricopharyngeal web, folate, selenium, zinc deficiency, corrosive injury to the esophagus, achalasia cardia, human papilloma virus infection, particularly serotype 16 and 18, Tylosis, history of head and neck (oral cavity, pharynx, larynx) or lung cancer, celiac disease, esophageal lichen planus, esophageal epidermoid metaplasia, Peutz-Jeghers syndrome and PTEN hamartoma tumor syndromes (e.g., Cowden syndrome) [31,32,33,34].

Symptoms: Patients generally present with progressive dysphagia and weight loss. Initially, the dysphagia is only to solid food, but as the disease progresses, the patient develops both solid and liquid food dysphagia. In the advanced stage, patients may present with hematemesis or melena due to ulceration of the tumor. If the cancer invades the recurrent laryngeal nerve, patients may present with cough or hoarseness of voice [35].

Diagnosis: Imaging studies, such as a barium swallow and CT scan, may help detect the tumor in the esophagus. Upper endoscopy with biopsy is the gold standard in establishing the diagnosis [36].

Management: involves a multimodality approach, depending on the tumor stage and host factors [37].

Early-stage cancer: Endoscopic resection is the treatment of choice if the tumor is confined to the mucosa (T1, N0, M0). Esophagectomy is recommended if there is submucosal invasion of cancer.

Locally advanced disease (T2 or higher, N+. M0): Neoadjuvant chemoradiotherapy (CRT) therapy followed by esophagectomy is recommended by the American Society of Clinical Oncology. Improved survival has been observed with the use of carboplatin and paclitaxel in the neoadjuvant regimen. Definitive CRT is considered when patients are not surgical candidates or do not wish to undergo esophagectomy [38].

Metastatic or unresectable disease: The standard of care is definitive CRT. Cisplatin and 5-fluorouracil (CF) are commonly used chemotherapeutic agents. A combination of chemotherapy and checkpoint inhibitors (Ipilimumab or Nivolumab) has shown a survival benefit in metastatic disease [39].

## 9. Eosinophilic Esophagitis (EoE)

This is a food-related, chronic, immune-mediated inflammatory disorder of the esophagus that affects both children and adults and is widely distributed throughout the world. It is most commonly seen between the ages of 30 and 50. The incidence of EoE is approximately 1 in 10,000 new cases per year. The prevalence of EoE in the United States is approximately 142.5 cases per 100,000 people [40]. It is 3 times more common in males than in females. EoE is a patchy disease and can affect any part of the esophagus [41]. Multiple factors play into the pathogenesis of EoE. These include genetic predisposition, exposure to food allergens, immune response, and epithelial barrier dysfunction. A genome-wide association study (GWAS) found the thymic stromal lymphopoietin (TSLP) gene at the 5q22 locus and the calpain 14 (CAPN14) gene at the 2p23 locus. These genes doiare upregulated in patients with EoE. CAPN14 is an intracellular protease that impairs epithelial barrier function in patients with EoE [42]. TSLP is expressed by epithelial cells and can activate dendritic cells, i.e., antigen-presenting cells. When food allergens come in contact with esophageal epithelial cells, the dendritic cells process those antigens. CD4 cells differentiate into type 1 T helper (Th1) cells and type 2 T helper (Th2) cells. Th1 cells secrete IFN-Υ and TGF-β. They are responsible for esophageal remodeling, fibrosis, stricture, and dysmotility. Th2 cells secrete interleukin (IL)-4, IL-5, and IL-13. These cytokines stimulate the secretion of eotoxin-3 and reduce filaggrin secretion. Eotoxin-3 is a potent chemoattractant of eosinophils, which cause secretion of cytokines, chemokines, macrophage migration inhibition factor (MMIF), and TNF, leading to inflammation. Decreased filaggrin reduces the epithelial barrier and increases epithelial permeability [43]. Although the exact mechanism of EoE development is unknown, exposure of the esophagus to food and aeroallergens in genetically predisposed individuals initiates the process of EoE. Foods most commonly implicated in EoE include milk, eggs, wheat, soy, peanuts, beans, rye, and beef. EoE can be associated with other type 2 inflammatory diseases, such as allergic rhinitis, bronchial asthma, IgE-mediated food allergies (cow’s milk is a major food allergen), and atopic dermatitis. EoE is a progressive disease. Delays in diagnosis or treatment can lead to the development of an esophageal stricture.

Symptoms: The most common symptoms of EoE in adults are dysphagia and food impaction [44]. EoE can be diagnosed in 12 to 23% of patients undergoing endoscopy for dysphagia [45]. 50% of adult patients with food impaction who undergo upper endoscopy are diagnosed with EoE [46]. Other symptoms of EoE include heartburn and chest pain.

**Figure 1:**
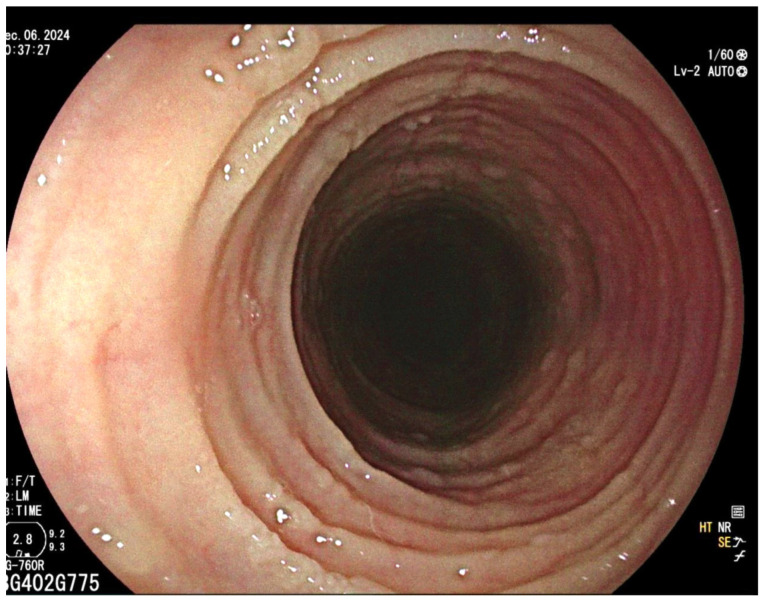
EoE showing rings and furrows.

Diagnosis: The diagnosis of eosinophilic esophagitis (EoE) is established when the patient presents with signs of esophageal dysfunction, such as dysphagia. This diagnosis is supported by the presence of specific endoscopic features, which may include edema, rings, exudates, linear furrows, white plaques, and strictures (Figure 1). Additionally, the diagnosis requires evidence of esophageal eosinophilia, defined as having at least 15 eosinophils per high-power field (HPF) in at least one of six biopsies taken from the proximal, mid, and distal esophagus. It is also essential to exclude other potential causes of esophageal eosinophilia, such as gastroesophageal reflux disease (GERD), achalasia, pill esophagitis, drug hypersensitivity reactions, Crohn’s disease involving the esophagus, as well as fungal, viral, or parasitic infections, graft-versus-host disease, pemphigus, allergic vasculitis, and hypereosinophilic syndrome [47].

Management: The management of EoE needs a multidisciplinary approach. Drugs, diet, and dilation are the three primary modalities of therapy. The drugs used in EoE include PPI, topical steroid preparations (fluticasone and budesonide), and dupilumab. High-dose PPI (Omeprazole 40 mg twice daily or equivalent) is considered the initial treatment of choice for 8 to 12 weeks, given its availability, low cost, and favorable safety profile. Symptomatic improvement occurs in 40 to 50% of patients, and histologic improvement (<15 eosinophils per HPF on esophageal biopsy) occurs in 41.7% of patients [48]. If the patient responds to PPI, a maintenance dose of Omeprazole 40 mg or equivalent is given once daily. PPI blocks IL-4/IL-13 mediated STAT6 dependent expression of exotoxin-3 and reduces esophageal eosinophilic infiltration [49]. PPI also restores esophageal mucosal barrier function by increasing filaggrin expression [50]. The topical steroid fluticasone comes in an inhaler. It is puffed directly into the mouth without breathing and then dry swallowed. 880 micrograms (mcg) are given twice daily for 8 weeks as an induction dose, followed by 440 mcg twice daily as a maintenance dose. It is best administered postprandially or at bedtime, with a 30- to 60 min wait after each dose to avoid consuming any food or water. Budesonide oral suspension 2 mg twice daily is administered as an coinduction dose for 12 weeks, followed by 1 mg twice daily as a maintenance dose. Clinical improvement occurs in 50–80% of cases, and histologic remission occurs in 64.9% of cases. Dupilumab (a fully human monoclonal antibody against IL-4 and IL-13) 300 mg subcutaneously weekly is considered for patients who are resistant to PPI or topical steroid therapy or have multiple atopic conditions. A Phase 3 trial showed that dupilumab therapy significantly improved symptoms, quality of life, and endoscopic and histologic outcomes in EoE patients [51]. Food elimination diet (FED) is an effective therapy in the management of EoE, but it is less palatable and not well accepted by patients. An elemental diet is more efficacious than an empirical FED and a skin allergy-tested diet. In an elemental diet, food allergens are removed by using an amino acid formula. When an elemental diet is given for 6 weeks, symptomatic and histologic improvement occurs in more than 90% of cases [52]. 6-food (cow’s milk, wheat, egg, seafood, nuts, soy/legumes), 4-food (cow’s milk, wheat, egg, soy/legumes), 2-food (cow’s milk, wheat), and 1-food (cow’s milk) elimination diets induce histologic remission in 40 to 70% of cases. If dietary therapy is advised, 1-FED or 2-FED should be chosen first in consultation with a dietitian. Symptomatic, endoscopic, and histologic assessments should be performed after 6 to 8 weeks. Endoscopic dilation is indicated for symptomatic esophageal stricture (esophageal diameter < 10 mm), long-segment narrowing, and narrow-caliber esophagus. This modality of treatment improves dysphagia and quality of life but does not reduce esophageal eosinophilia. Balloon dilator is ideal for short (1 to 2 cm) stricture, and Savary dilator is good for long-segment narrowing and narrow-caliber esophagus. Esophageal luminal diameter should be 16 mm or more to relieve dysphagia. A systematic review revealed a risk of perforation in fewer than 1% of cases in this patient group [53].

## 10. Esophageal Leiomyoma

This occurs in less than 1% of all esophageal neoplasms. It is the most common benign esophageal mesenchymal tumor. The most common site of esophageal leiomyoma is the distal two-thirds of the esophagus. It is a slow-growing tumor with a low chance of malignancy. It is a well-circumscribed spindle cell tumor arising from the muscularis propria or muscularis mucosae of the esophagus. Sometimes, it can grow to a large size, exceeding 10 cm, and is then referred to as a giant leiomyoma of the esophagus [54].

Symptoms: Most esophageal leiomyomas are small (<5 cm), and patients remain asymptomatic for an extended period. Large leiomyomas can cause dysphagia or chest pain. They are more common in males than in females (2:1), with the usual age of presentation ranging from 20 to 50 years.

Diagnosis: Esophageal leiomyoma can be detected through imaging studies, such as a chest X-ray, barium swallow, CT scan, or MRI scan. A chest X-ray may show a posterior mediastinal mass. A partial luminal filling defect is seen on a Barium Swallow. A CT scan may show a hypodense or iso-dense intramural smooth, eccentric lesion of varying size arising from the muscular propria or muscularis mucosae. MRI may reveal the lesion as slightly hyperintense [55]. Esophagoscopy can find an esophageal leiomyoma as a subepithelial lesion. EUS with fine needle aspiration (FNA), fine-needle biopsy (FNB), or mucosal incision–assisted biopsy (MIAB) is the investigation of choice. The subepithelial lesion is seen as homogeneous and hypoechoic, arising from the muscularis propria or muscularis mucosae [56]. Histology is characterized by the proliferation of interlacing bundles of bland, smooth muscle cells, which have long, narrow nuclei but few mitotic figures. They stain positive for Desmin and alpha-smooth muscle actin but negative for CD34, CD117, and S100 protein, distinguishing them from GIST, glioma, Schwannoma, neurofibroma, and melanoma [57].

Management: If the patient is asymptomatic, the patient can be observed clinically without intervention. Symptomatic and large (>5 cm) leiomyomas need to be enucleated. Enucleation can be performed surgically or endoscopically. Thoracoscopic enucleation or laparoscopic transhiatal enucleation is an effective minimally invasive surgical approach for treating esophageal leiomyoma. In selected cases, submucosal tunneling endoscopic resection (STER) is being increasingly performed with similar efficacy to surgical enucleation [58]. A very large esophageal leiomyoma may need esophageal resection [59].

## 11. Esophageal GIST

This is rare, accounting for less than 5% of all GISTs. The usual site is the distal or middle third of the esophagus. It is a subepithelial, well-circumscribed tumor that arises from interstitial cells of Cajal in the muscularis propria or muscularis mucosae. It is a slow-growing spindle cell tumor with potential for malignancy. It equally affects males and females. The average age of diagnosis is 60 years. The risk stratification depends on tumor size and mitotic index. Tumor size greater than 5 cm and a mitotic count greater than 5 per 50 high-power field indicate a high-risk group [60].

Symptoms: Patients with small esophageal GIST typically remain asymptomatic. Occasionally, they are identified during imaging studies (like a CT scan) or an upper endoscopy. As the tumor grows bigger, patients may present with dysphagia, chest pain, or upper gastrointestinal bleeding.

Diagnosis: CT with contrast can identify GIST as an intramural endophytic or exophytic lesion arising from the muscular layer of the esophagus. Upper endoscopy shows a well-circumscribed spherical or hemispherical smooth subepithelial lesion in the esophagus. Endosonographically, the lesion appears as inhomogeneous and hypoechoic, arising from the muscularis propria or rarely from the muscularis mucosae. Tissue acquisition is performed by EUS-guided FNA or true-cut biopsy (TCB). Histopathology shows spindle cell, epithelioid, or mixed morphology, and immunohistochemical staining is positive for KIT (CD117), CD34 (expressed in 75% of cases), DOG1 (expressed in 65 to 100% of cases), and PDGFRA (expressed in 15% of cases) [61]. Definitive diagnosis of GIST is established when histology is in concordance with immunohistochemical staining.

Management: Symptomatic esophageal GIST can be managed surgically or endoscopically. Surgical enucleation is the treatment of choice for small GISTs, while esophagectomy is recommended for larger GISTs. Small, low-risk GIST can be treated with STER, which is now considered an effective and safe treatment option. For high-risk cases, adjuvant therapy with the tyrosine kinase inhibitor (TKI) imatinib is recommended after resection of GIST for at least 3 years. Imatinib is also recommended for the treatment of unresectable, recurrent, and metastatic GIST as a standard of care. Molecular profiling should be performed before initiating therapy, as patients with PDGFRA mutations are resistant to imatinib therapy. In patients without PDGFRA mutation, imatinib is the first-line therapy. In imatinib-resistant or intolerant GIST, sunitinib (a multitargeted TKI) is the next treatment option. Regorafenib (another multitargeted TKI) should be given to patients previously treated with imatinib and sunitinib. In cases of large GIST, neoadjuvant therapy with TKI is recommended for a duration of 6 to 12 months preoperatively to downsize the GIST, facilitating resection or preserving organ function [62].

## 12. Traction Esophageal Diverticulum (TED)

This accounts for 15% of all esophageal diverticula. It is a true mid-esophageal diverticulum, i.e., it involves all the layers of the esophageal wall and is caused by traction from mediastinal inflammation. Granulomatous disorders, such as sarcoidosis, histoplasmosis, and tuberculosis, can lead to mediastinal lymphadenitis and subsequent fibrosis. The fibrosis can pull the esophageal wall outward, forming a broad-mouthed, triangular, or polygonal diverticulum. The mechanism of formation of TED is different from that of pulsion diverticuli (Zenker diverticulum, epiphrenic diverticulum) which are formed by increased intraluminal pressure because of esophageal dysmotility and involve only mucosa and submucosa (pseudo-diverticuli).

Symptoms: Most patients with TED are asymptomatic and are diagnosed incidentally during imaging studies or endoscopy. Generally, patients are elderly with prior history of granulomatous disorders. Rarely, patients may present mild dysphagia, regurgitation, or chest discomfort.

Diagnosis: A barium esophagogram is the gold standard for diagnosing TED. CT chest shows a well-defined, saccular outpouching arising from the mid-esophagus near the carina or mainstem bronchus. Adjacent mediastinal fibrosis can also be visible. Upper endoscopy can confirm the diagnosis.

Management: No treatment is needed in asymptomatic cases. Surgical intervention like diverticulectomy with or without myotomy should be considered in symptomatic cases [63].

## 13. Esophageal Lichen Planus (ELP)

This is a rare, chronic inflammatory condition of the esophagus, primarily seen in the proximal or mid esophagus. Middle-aged and elderly females are commonly affected. It is often associated with oral lichen planus, sometimes with cutaneous or genital lichen planus. The exact pathogenesis of ELP is unknown. The current understanding is that it is a T-cell-mediated autoimmune disease. Certain environmental factors like virus (particularly hepatitis C virus, COVID-19 virus), medications (like antimalarials, non-steroidal anti-inflammatory drugs, thiazide diuretics, angiotensin converting enzyme inhibitors, beta-blockers, quinidine, and anti-tumor necrosis factors), and dental restoration materials (like copper, mercury and gold) alter the epithelial cell antigens on the mucosal surface of esophagus. This alteration triggers cytotoxic T-lymphocytes, which attack the epithelial cells and cause apoptosis, resulting in a characteristic appearance. The immune response becomes chronic, resulting in chronic inflammation with subsequent consequences, such as stricture and cancer [64]. Sometimes, genetic predisposition plays a role in the development of ELP.

Symptoms: Patients can be asymptomatic, but 80% of patients with ELP may present with dysphagia. Other symptoms include odynophagia, heartburn, and regurgitation.

**Figure 2:**
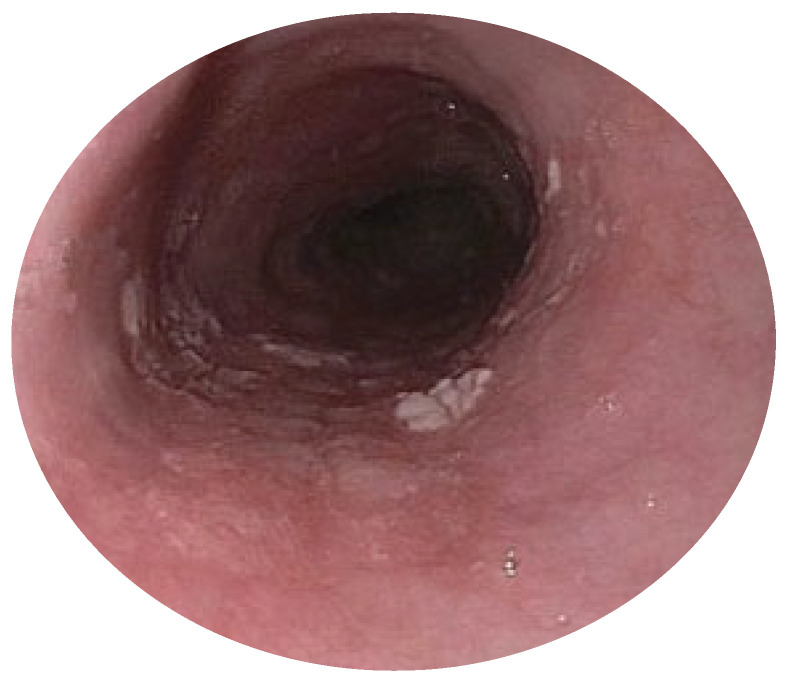
Esophageal lichen planus.

Diagnosis: ELP is generally suspected based on endoscopic appearance and then confirmed by taking a biopsy and examining the histology. The endoscopic appearance includes a white, lacy appearance (Figure 2), loss of vascular pattern, hyperkeratosis, friability, desquamation, white plaques, trachealization, erosions, and strictures [65]. Histology shows basal cell hydropic degeneration and apoptosis (Civatte bodies), dense T-cell infiltration of the lamina propria, hyperkeratosis, dyskeratosis, and fibrin deposition along the basement membrane seen on direct immunofluorescence [66].

Management: Currently, there is no accepted guideline for the treatment of ELP. Topical corticosteroids, such as budesonide oral suspension (3 mg twice daily) or inhaled fluticasone (880 μg twice daily), have been shown to cause clinical and/or endoscopic improvement in 62–74% of cases [67,68]. Oral prednisone 40 to 60 mg daily for 4 to 6 weeks should be given if the patient does not respond to a topical steroid. Other immunosuppressants, such as intralesional triamcinolone acetate, tacrolimus, and cyclosporin, have been shown to improve symptoms in patients who are refractory to oral prednisone [69]. Dilation of stricture secondary to lichen planus is an effective treatment for symptomatic improvement. ELP is a premalignant condition, although the risk of transformation into esophageal squamous cell carcinoma per year is unknown. Upper endoscopy is recommended by some authors to be performed every 1 to 2 years [66].

## 14. Esophageal Stricture (ES)

The prevalence of ES in the United States ranges from 1 in 100 to 1 in 1000 patients. It was 203.14 cases/100,000 people in 2021, as per MarketScan databases [70]. ES can affect patients of any age, but patients 75 years or older are more commonly affected. Benign strictures are more common in the younger population, whereas malignant strictures are more widely seen in the older population. White persons are 10 times more commonly affected by peptic strictures than Asian or black persons. Although the incidence of different ES increases with age, children and young adults are more widely affected by strictures secondary to eosinophilic esophagitis and caustic esophagitis.

Etiology: Peptic esophageal stricture due to gastroesophageal reflux disease (GERD) is the most common cause, accounting for 70–80% of adult cases [71]. Over the last two decades, the prevalence of ES has markedly increased in newly diagnosed eosinophilic esophagitis (EOE) patients (8% in 2006 to 54% in 2019 [72]. Another benign cause of ES is caustic stricture secondary to intentional or accidental ingestion of caustic substances. One-third of patients with severe caustic esophageal injury (grade IIB or III) generally develop stricture two months after the injury. Still, it can vary from two weeks to many years [73]. Radiation-induced ES occurs when thoracic external beam radiation therapy with a mean dose of >50 Gy is administered for various malignancies like lung cancer, head and neck cancer, esophageal cancer, thymic cancer, malignant mesothelioma, and lymphoma. When the thoracic radiation dose is 50 Gy or less, symptomatic ES occurs in less than 2% of cases compared to approximately 15% of cases when treated with 60 Gy [74]. Iatrogenic ES can occur following ablation of Barrett’s esophagus with different modalities of treatment: (a) radiofrequency ablation (5 to 10% of cases), (b) liquid nitrogen cryotherapy ablation (3% of cases), (c) carbon dioxide cryotherapy ablation (1.5% of cases), and (d) photodynamic therapy (34% of cases) [75,76,77,78]. Endoscopic submucosal dissection (ESD) of early-stage esophageal cancer (T1a lesion) can also have a high chance of developing ES (70 to 80% if the resection is >60% of esophageal circumference) [79]. Post-peroral endoscopic myotomy (POEM) ES is uncommon (1.1 per 10,000 person-years) when treated for achalasia [80]. The incidence of anastomotic stricture following Ivor-Lewis esophagectomy for esophageal cancer may vary from 9.1% to 46% [81]. Drug-induced ES has been reported to be caused by a variety of medications, which include alendronate, potassium chloride, tetracycline, ferrous sulfate, non-steroidal anti-inflammatory drugs, quinidine, phenytoin, and ascorbic acid [82]. Prolonged use of the nasogastric tube can cause ES. Rarely, ES can be caused by Crohn’s disease, graft versus host disease (GVHD), pemphigus vulgaris, pemphigoid, esophagitis dissecans superficialis associated with celiac disease, collagen vascular diseases such as SLE or scleroderma, tuberculosis, and Plummer-Vinson syndrome [83].

Pathophysiology: Benign ES generally occurs secondary to the long-standing inflammatory process in the esophagus wall, irrespective of the underlying etiology. Chronic inflammation leads to intramural fibrosis, which subsequently narrows the esophageal lumen. Malignant ES usually occurs due to adenocarcinoma (involving the lower third of the esophagus) or squamous cell carcinoma of the esophagus (involving the middle or upper third of the esophagus). Rarely, pulmonary or mediastinal malignancy or enlarged mediastinal lymph node can affect the esophagus, leading to stricture.

Classification: ES are broadly classified into simple strictures and complex strictures. Simple structures are characterized by short (<2 cm), symmetrical, focal, and straight strictures (luminal diameter > 12 mm) with smooth surfaces and borders. Common causes of simple esophageal strictures include peptic esophageal strictures and EoE-induced strictures. Complex esophageal strictures are long (>2 cm), asymmetrical or angulated, and narrower (<12 mm) with uneven surfaces and borders [84]. Caustic stricture, radiation-induced stricture, anastomotic stricture, and post-ESD strictures are usually complex esophageal strictures and are generally refractory and recurrent [85]. The stricture is labeled as refractory when a dysphagia score of 2 or more remains persistent due to unsuccessful dilation of the esophageal stricture to 14 mm despite multiple dilations (>5 sessions) at two-week intervals. About 10% of benign esophageal strictures are refractory. Recurrent stricture is suspected when the patient becomes symptomatic due to the unsuccessful maintenance of a satisfactory luminal diameter for four weeks, despite achieving a target diameter of 14 mm once [86]. ES can also be classified based on radiologic and endoscopic assessment of length, internal diameter, and difficulty in dilation of the stricture [87,88].

Type I or mild stricture: length < 2 cm, 9 to 12 mm diameter, and uncomplicated to dilate.

Type II or moderate stricture: length 2 to 4 cm, diameter 5 to 8 mm, and requires more aggressive dilation.

Type III or severe stricture: length > 4 cm, diameter < 5 mm, refractory to simple dilation, and may require advanced interventions like stricturotomy, stenting, or esophageal resection.

A multivariable analysis performed at Mayo Clinic recently showed a strong predictive model associated with a higher risk of refractory benign ES if the stricture length is ≥2cm, the diameter is ≤7 mm and based on the proximal location of the stricture or diffuse stricture [89].

Symptoms: Dysphagia, i.e., difficulty swallowing food or delay in the passage of food through the esophagus, is ES’s most common symptom. Although the standard diameter of the esophageal lumen is 30 mm, solid food dysphagia occurs when it narrows beyond 12 mm. With the progression of stricture, patients develop difficulty swallowing a semisolid to liquid diet. Ogilvie dysphagia score is shown below [90]. Sometimes, patients may experience painful swallowing (odynophagia), coughing or gagging, drooling, or regurgitation during the swallowing process.

Ogilvie dysphagia score (range 0–4)

- 0 = no dysphagia
- 1 = normal diet, avoiding certain foods
- 2 = semisolid food only
- 3 = liquids only
- 4 = complete dysphagia, even for liquids

Patients may have other associated symptoms due to underlying causes of ES. Patients with GERD can have a history of long-standing or uncontrolled heartburn in the past. Patients with EoE may give a history of prior food bolus impaction, bronchial asthma, atopic dermatitis, or chronic spontaneous urticaria. Patients with underlying malignancy may suffer from anorexia and weight loss. A history of prior surgery, endoscopic intervention, radiation, or medication can give essential clues to the diagnosis of esophageal stricture. Irrespective of the etiology, ES causes weight loss due to inadequate food intake and poor quality of life.

Diagnosis: Barium Swallow and upper endoscopy are critical tests in diagnosing ES. A barium swallow is particularly valuable in the diagnosis of suspected complex strictures. Upper endoscopy is essential to find out the underlying cause of ES; biopsy should be taken from all strictures, and it also has therapeutic options. Sometimes, it is difficult to differentiate benign from malignant ES. In cases of suspected malignant stricture with a negative mucosal biopsy, endoscopic ultrasound (EUS) with fine needle aspiration (FNA) is a crucial diagnostic tool [91]. Both EUS and computerized tomography (CT) help evaluate the depth of invasion of malignant ES [92].

Management: The main principle of treating ES is symptomatic improvement with relief of dysphagia. The treatment of symptomatic ES depends on whether it is a benign or malignant lesion. The treatment modalities for benign ES include drugs, dilation, stricturotomy, and surgery.

Drugs: High-dose, long-term proton pump inhibitors (PPIs) should be administered for peptic esophagitis [93]. Patients with EoE should continue to receive PPI, topical steroids, or dupilumab before and after pre-dilation of the esophagus [94]. PPI should also be given after endoscopic resection or ablation to reduce the formation of ES [95]. Other medications can be helpful depending on the cause of stricture like in case of Crohn’s stricture—steroid, biologics, immunomodulators; in GVHD, pemphigus vulgaris and pemphigoid—topical steroid and systemic immunosuppression [96]; esophagitis dissecans superficialis associated with celiac disease—gluten-free diet [83]; and collagen vascular diseases—systemic steroid and immunosuppressive therapy [97].

Dilation: Endoscopic dilation of a benign ES (either by balloon or bougie) is considered the first-line treatment to improve dysphagia. Bougie (push-type dilators: wire-guided Savary-Gilliard dilator or blind Maloney dilator) dilators exert radial and longitudinal force, whereas through the scope (TTS), pneumatic or balloon dilators only exert radial force. Maloney dilatators are suitable for distal single strictures with a luminal diameter of 10 mm or more. In the case of mid-esophageal or proximal ES, a pneumatic or Savary-Gilliard dilator should be used. Fluoroscopy should be used in case of complex ES. It can help the advancement of the guidewire and the balloon catheter through the stricture. It can also assess the length and diameter of the stricture before and after dilation, as well as the presence of any fistula and post-dilation extravasation of contrast. The ultimate goal is to achieve a luminal diameter of 14 mm or more so the patient can tolerate a regular diet. It is recommended to pass no more than 3 dilators of progressively larger diameter (with an increment of 1.5 mm) per session (rule of three) in the presence of moderate to severe resistance to avoid adverse effects like perforation of the esophagus [98]. The risk of perforation is 0.1 to 0.4% during ES dilation [99]. Dilation is adequate in at least 90% of patients with simple ES [100]. Intralesional steroid injection should be considered in refractory and recurrent ES, as it can decrease collagen and fibrous tissue deposition into the stricture. It has been demonstrated that four-quadrant injections of steroid (triamcinolone acetonide, 40 mg/mL, 0.2 mL to 0.5 mL aliquots) into the stricture, either before or after dilation, can improve the post-dilation diameter and delay the need for subsequent dilation [71].

Stenting: If the stricture is refractory, recurrent, and/or non-responsive to steroid injection, temporary (at least 6–8 weeks, maximum 3 months) placement of a fully covered self-expanding metal stent (FCSEMS) made of mostly nitinol or a self-expanding plastic stent (SEPS) made of silicone with polyester braid or a biodegradable esophageal stent (BDS) should be considered [101]. FCEMS and SEPS placement complications include stent migration (in 4% to 36% of cases), tissue hyperplastic reaction, gastroesophageal reflux, fistula formation, Globus sensation, chest pain, fever, and bleeding [102]. The stent migration rate can be reduced with endoscopic suturing (15.9% lower than without intervention) or fixation with an over-the-scope clip (OTSC) system (8.3% with fixation vs. 35.4% without fixation) [103]. BDS is made from polydioxanone and can provide constant radial force for 4 to 5 weeks without tissue overgrowth. Then, it slowly dissolves over 11 to 12 weeks due to progressive hydrolysis and degradation. As a result, the BDS does not need to be removed endoscopically [104]. Long-term relief of dysphagia in refractory benign ES can be achieved in 30% to 40% of cases with the temporary placement of an esophageal stent [105].

Endoscopic stricturotomy/strctureplasty/electroincision is an endoscopic procedure in which the fibrous tissue under the mucosa at the most prominent part of the esophageal stricture is carefully cut in layers with a needle knife in a circumferential manner (electrocautery technique). Endoscopic ultrasound with a linear probe can be performed before this procedure to evaluate the wall thickness of the stricture [106]. An advanced, proficient endoscopist typically performs this procedure, which is beneficial for refractory strictures, short-segment non-angulated fibrotic strictures (<1 cm), and anastomotic strictures [107]. There are few case reports in which refractory complex ES was treated by endoscopic sphincterotomy [108].

Surgery is considered as the last resort when endoscopic therapy fails. A laparoscopic or open esophageal resection is performed for distal ES. Partial or complete fundoplication is performed simultaneously in the case of peptic ES [109]. For mid or proximal ES, particularly in corrosive strictures, transhiatal esophagectomy with gastric pull-up and cervical anastomosis is considered safe. As colonic transposition changes gastrointestinal anatomy and carries increased morbidity and mortality, it is performed when gastric reconstruction is not suitable [110]. Collis gastroplasty with Belsey herniorrhaphy is considered when the esophagus becomes excessively short due to stricture [111]. ES bypass with gastric pull-up or colonic interposition can be performed in cases of long-segment strictures, particularly those involving the upper esophagus, when resection is not feasible [112].

Malignant ES is managed by stent placement, chemoradiation, or surgery. If the patient’s life expectancy is less than 3 months or if the malignancy is inoperable, palliative self-expanding metal stent (Wallstent, Ultraflex stent, or Niti-S) placement can expand the esophageal lumen and rapidly improve the patient’s dysphagia. Esophageal brachytherapy is mainly used in patients with a life expectancy of more than 3 months [113]. Pre-operative chemoradiation followed by surgery/esophageal resection is performed in case of resectable or early malignant ES [99].

## 15. GERD

This is a common disease in which reflux of gastric contents into the esophagus results in symptoms and/or complications [114]. The prevalence of GERD in the United States is about 20% [115]. But there are a few risk factors like obesity, smoking, and genetic predisposition, which can increase the chance of developing GERD. The pathophysiology is multifactorial. Inappropriately frequent and prolonged relaxation of the lower esophageal sphincter (LES) is the primary mechanism of GERD. Other factors include low LES pressure, dysfunction of crural diaphragm, hiatus hernia, delayed gastric emptying, and impaired esophageal clearance [116].

Symptoms: Heartburn and acid regurgitation are the classic symptoms of GERD. Non-cardiac retrosternal chest pain is another frequent symptom of GERD. Sometimes, patients may present with extra-esophageal symptoms, such as chronic cough, wheezing, hoarseness of voice due to posterior laryngitis, and globus sensation (i.e., a feeling of a lump in the throat). Patients may also report dysphagia, odynophagia and upper gastrointestinal bleeding.

Complications: GERD decreases patients’ health-related quality of life. Esophageal complications of GERD include erosive esophagitis, esophageal ulcer, esophageal stricture, and Barrett’s esophagus (i.e., standard lining of stratified squamous epithelium in the distal esophagus is replaced by a metaplastic simple columnar epithelium with mucin-secreting goblet cells, also called specialized intestinal metaplasia) that may lead to esophageal adenocarcinoma [117]. Currently, endoscopic staging of esophagitis is performed using the Los Angeles (LA) classification, as follows: [118].

**Figure 3:**
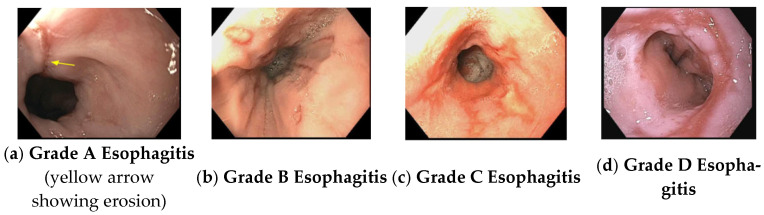
LA classification of esophagitis.

LA classification (Figure 3):

Grade A: 5 mm or less erosion in mucosal folds
Grade B: Erosion > 5 mm in mucosal folds but no continuity between folds
Grade C: Mucosal erosion is continuous between 2 or more mucosal folds but involving less than 75% of the circumference.
Grade D: All-around mucosal erosion involving more than 75% of the esophageal circumference.

Diagnosis: is based on clinical presentation, response to acid suppressant therapy, endoscopic evidence and objective evidence of pathological acid reflux. Although heartburn and regurgitation are suggestive of GERD, they are neither sensitive nor specific enough to establish the diagnosis of GERD in isolation. Again, positive or negative response to proton pump inhibitor (PPI) therapy cannot confirm or exclude the diagnosis of GERD, respectively. PPI non-responders should have upper endoscopy. Endoscopic evidence of GERD includes LA grade B, C, or D erosive esophagitis (Figure 3), long-segment (≥3 cm) Barrett’s esophagus, and peptic esophageal stricture. An ambulatory esophageal pH study is not required in these cases. In the absence of positive endoscopic findings, ambulatory pH or pH-impedance testing, preferably performed off PPI therapy, should be conducted to evaluate abnormal esophageal acid exposure (an acid exposure time of >6 min is diagnostic) [119]. Patients who present with non-cardiac chest pain should be evaluated by endoscopy and ambulatory pH study. Patients with alarm symptoms (dysphagia, anorexia, weight loss) or multiple risk factors for Barrett’s esophagus (chronic GERD with weekly symptoms for 5 years or more, age more than 50 years, white race, male sex, central obesity, smoker, hiatus hernia and family history of Barrett’s esophagus or esophageal adenocarcinoma) should be evaluated by upper endoscopy first [120]. High-resolution manometry (HRM) should also be performed in patients who are unresponsive to PPI therapy and have a negative pH or pH-impedance study, particularly in those with non-cardiac chest pain [121]. An esophageal impedance-pH study should be performed on PPI therapy if patients with an established diagnosis of GERD have symptoms refractory to PPI therapy.

Management: includes lifestyle modification, medical, surgical, and endoscopic therapy for the treatment of GERD.

Lifestyle modification: The most substantial evidence of benefit includes avoiding food intake within 3 h of bedtime, elevating the head of the bed by 6 to 8 inches, and weight loss in individuals who are overweight or have recently gained weight. Other recommendations include avoiding tobacco products/smoking cigarettes, drinking alcohol, high-fat and spicy food, caffeine, chocolate, peppermint, carbonated beverages, citrus juices, onions, tomato paste, or any trigger food. Medications (such as certain calcium channel blockers, tricyclic antidepressants, anticholinergics, theophylline, sildenafil, progesterone, and benzodiazepines) that can lower the LES should also be avoided. The patient should also engage in stress reduction measures. Chewing gum may help salivary secretion, which can neutralize acid reflux [122].

Medication: Patients with classic symptoms of GERD without any alarm features should be given empiric PPI therapy once a day, 30 to 60 min before a meal, for 8 weeks. Attempt should be made to discontinue PPI therapy in patients who respond to the empiric PPI trial. Patients with LA grade C or D esophagitis should be offered PPI maintenance therapy indefinitely or anti-reflux surgery. For maintenance therapy, PPI should be given at the lowest effective dose that can control GERD symptoms and maintain healing of reflux esophagitis. Long-term PPI therapy outweighs the theoretical risks and side effects. Patients with heartburn due to non-erosive reflux disease (NERD) should be given intermittent or on-demand PPI therapy. Baclofen is not recommended in patients without any objective evidence of GERD. Patients with typical GERD symptoms and extra-esophageal manifestations should be given PPIs twice daily for 8 to 12 weeks before performing endoscopy. An esophageal pH study should be performed in patients with extra-esophageal manifestations who do not exhibit classic symptoms of GERD before initiating PPI therapy. PPI therapy should be discontinued in patients with refractory classic symptoms of GERD but have normal pH study off PPI therapy, and normal pH/impedance study on PPI therapy.

Surgical therapy: Anti-reflux surgery (laparoscopic fundoplication) is indicated in patients with persistent symptoms despite optimal dose of PPI therapy, LA grade C or D esophagitis and large hiatus hernia. Roux-en-Y gastric bypass surgery should be considered in morbidly obese patients with GERD who are candidates for bariatric surgery.

Endoscopic therapy: Transoral incisionless fundoplication (TIF) is an effective endoscopic procedure in which anti-reflux valve at the gastroesophageal junction is reconstructed. It should be considered in patients who have troublesome heartburn or regurgitation despite PPI therapy. Still, they do not have LA grade C or D esophagitis or hiatus hernia > 2 cm and do not want anti-reflux surgery [123].

## 16. Barrett’s Esophagus (BE)

**Figure 4:**
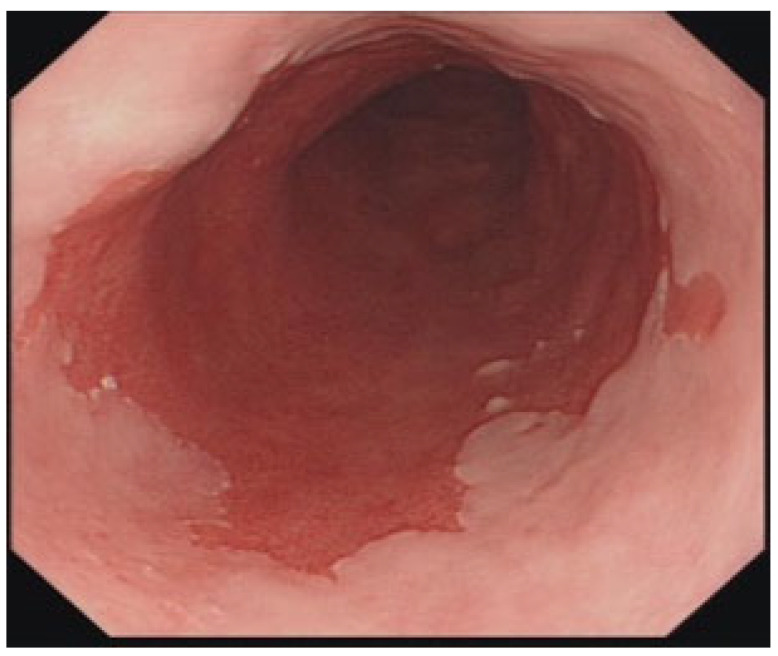
Barrett’s esophagus.

This is a condition in which the usual lining of stratified squamous epithelium in the distal esophagus is replaced by a metaplastic simple columnar epithelium with mucin-secreting goblet cells, also called specialized intestinal metaplasia. The columnar mucosa is easily recognized endoscopically by its salmon-pink color (Figure 4). Currently, in the United States, Barrett’s esophagus is defined as the extension of salmon-colored mucosa into the distal esophagus, 1 cm above the gastroesophageal junction, with histological confirmation of specialized intestinal metaplasia. The primary importance of Barrett’s esophagus lies in its premalignant condition, with a 0.5% annual risk of developing esophageal adenocarcinoma [124]. A simulation model confirmed by the US Surveillance Epidemiology and End Results (SEER) cancer registry data showed the estimated prevalence of BE in the general population to be 5.6% [125].

Pathogenesis and pathology: The exact pathogenesis is unknown. It is proposed to be a two-step process [126]. In the first step, there is a transformation of squamous epithelium into simple columnar epithelium. With continued acid exposure, there is injury to the distal esophageal mucosa and dilated intracellular spaces are seen histologically [127]. This change increases mucosal permeability, allowing particles up to 20 kDa to reach the esophageal stem cells in the basal layer of the esophagus. In the reparative process, the esophageal stem cells differentiate into columnar cells instead of squamous cells. The CDX gene plays a vital role in this transformation process. The cdx gene has been found in the Barrett’s mucosa and inflamed esophageal mucosa. It accelerates the progression from squamous to columnar metaplasia. The cdx promoter is activated on exposure to acid and bile [128]. So, duodenogastroesophageal reflux of bile may also play a role. Bile in the acidic pH becomes non-ionized and soluble. It can enter the esophageal epithelial cells and can cause toxic injury to mitochondria and cellular mutations [129]. The columnar mucosa extends up into the distal esophagus either as tongues or circumferentially. In the second step, the expression of intestinal genes enables goblet cells to populate the columnar epithelium, leading to intestinal metaplasia. Pathologically, non-dysplastic Barrett’s esophagus is characterized by columnar mucosa with mucin-filled blue goblet cells. The nuclei are regular in shape and size and aligned basally. The glands are well spaced and surrounded by plenty of lamina propria. In low-grade dysplasia (LGD), there are increased mitosis, cytological atypia, nuclear atypia with hyperchromatism and pleomorphism, crowding of glands with decreased intervening lamina propria, but nuclear polarity is preserved. In high-grade dysplasia, there is an increase in atypical mitosis, marked cytological changes, enlarged, hyperchromatic, and pleomorphic nuclei, with at least focal loss of polarity, and crowding of glands with almost complete loss of lamina propria [130].

Symptoms: Many Barrett’s esophagus patients are asymptomatic, and therefore the condition is under-diagnosed. BE is found in 10–15% of symptomatic GERD patients when their upper endoscopy is performed [131].

Diagnosis: Endoscopy with biopsy is the primary method for diagnosing Barrett’s esophagus. There is displacement of the squamocolumnar junction ≥ 1 cm above the GE junction. Endoscopic grading for Barrett’s esophagus was developed and validated in the Prague classification: the circumferential extent (Prague C) and maximal extent (including tongues) of salmon-colored mucosa (Prague M) are measured [132]. The Barretts’ segment can be tracked over time using this classification. Although patchy islands of Barrett’s mucosa are not mentioned in the Prague classification, they should be included in the endoscopy report. During endoscopy, at least eight random biopsies (four quadrant biopsies every 2 cm) should be taken from suspected Barrett’s mucosa to increase the yield of SIM. In cases of suspected short segment BE (<3 cm) where eight biopsies cannot be taken, at least four biopsies should be taken per cm of circumferential suspected Barrett’s mucosa, and one biopsy per cm in tongues of suspected Barrett’s mucosa. If there are any visible lesions, such as mucosal irregularities, nodules, or ulcers, targeted biopsies should be taken prior to random biopsies.

Screening: The American College of Gastroenterology (ACG) recommends screening for Barrett’s esophagus in males with chronic (>5 years) and frequent (weekly or more) symptoms of heartburn or acid regurgitation, as well as ≥2 of the following risk factors for Barrett’s esophagus or esophageal adenocarcinoma:

- Aged > 50 years
- Non-Hispanic white population
- Central obesity
- Present or history of smoking
- Confirmed family history of Barrett’s esophagus or esophageal adenocarcinoma in a first-degree relative.

Surveillance: The main objective of surveillance of patients with BE is to detect dysplasia or adenocarcinoma at an early stage so that effective treatment can be given. One study demonstrated that endoscopic surveillance detected Barrett’s esophagus-associated adenocarcinoma at a lower stage, resulting in improved survival [133]. The surveillance program begins with endoscopic biopsy of the Barrett’s mucosa, consisting of four-quadrant biopsies at 2 cm intervals in patients with non-dysplastic Barrett’s esophagus and four-quadrant biopsies at 1 cm intervals in patients with known dysplastic Barrett’s esophagus. High-definition white light endoscopy should be used. The ACG does not support routine use of advanced imaging except narrow-band imaging (NBI), which is a form of electronic chromoendoscopy. NBI may help in detecting dysplasia if targeted biopsies are taken from areas with irregular pattern [134].

Management: Depending on the histology of surveillance biopsies, further actions are taken:

- Non-dysplastic Barrett’s esophagus: endoscopic surveillance should be performed every 3–5 years as per ACG.
- Indefinite for dysplasia: patient should receive PPI therapy for 3–6 months. Repeat surveillance endoscopy with biopsies should then be performed. If the histology remains indefinite for dysplasia, endoscopic surveillance should be performed after 12 months.
- Dysplastic Barrett’s esophagus: any grade of dysplasia should be reviewed and confirmed by two pathologists, of whom at least one should be a gastrointestinal pathologist.
- LGD: endoscopic ablative therapy is the first choice in the absence of life-limiting comorbidity. Alternatively, endoscopic surveillance every 12 months is acceptable.
- HGD or intramucosal carcinoma (IMC): endoscopic ablative therapy is the treatment of choice in the absence of life-limiting comorbidity. Any nodule or mucosal abnormality should be removed by endoscopic mucosal resection (EMR).

Radiofrequency ablation (RFA) using the BARRX RFA system (Medtronic, California, USA) is considered the most effective and preferred modality of endoscopic ablation therapy. It is now the standard of care in the United States. RFA is safe and highly effective in eradicating both intestinal metaplasia and dysplasia in BE [135]. Post-RFA therapy, nausea, chest pain, and dysphagia can occur temporarily, but the esophageal stricture rate is approximately 1–6% [136].

Nodular Barrett’s esophagus:

EMR should be performed for both diagnostic and therapeutic purposes if there is any discrete nodule in the Barrett’s mucosa. If the histology of the nodule shows:

- HGD or IMC: the rest of the Barrett’s mucosa should be treated with endoscopic ablative therapy.
- Tumor invades lamina propria or muscularis mucosa (T1a) esophageal adenocarcinoma: endoscopic ablative therapy of the remaining BE is the next step.
- Tumor invades submucosa (T1b) esophageal adenocarcinoma: endoscopic ultrasound is usually carried out to evaluate the depth of tumor infiltration, and to biopsy local lymph nodes as there is a high rate of lymph node involvement in T1b esophageal adenocarcinoma [137]. Esophagectomy with consideration of neoadjuvant therapy is the preferred treatment. Endoscopic ablative therapy is an alternative if the carcinoma is superficial (sm1), well-differentiated without any lymphovascular invasion, or if the patient is a poor surgical candidate.
- T1b, sm2–3 esophageal adenocarcinoma: esophagectomy with consideration of neoadjuvant therapy is the preferred treatment.
- Irrespective of T1a or T1b esophageal adenocarcinoma, if there is any poor differentiation of carcinoma, lymphovascular invasion, or incomplete EMR, surgery with neoadjuvant therapy should be considered.

## 17. Esophageal Adenocarcinoma

This occurs most commonly in the lower third of the esophagus or near the gastroesophageal junction. The incidence of esophageal adenocarcinoma has increased significantly (about 5-fold) since 1970 making it the predominant subtype of esophageal carcinoma in the United States and Western countries. The risk factors include GERD, white race, male sex, tobacco smoking, obesity, and genetic factors [138]. The precursor lesion is BE, which grows through low-grade dysplasia and high-grade dysplasia.

Symptoms: Patients with early-stage adenocarcinoma generally remain asymptomatic. They are typically detected during surveillance of Barrett’s esophagus or upper endoscopy for GERD.

Patients with advanced disease generally present with dysphagia and weight loss [139]. Other symptoms include aspiration of saliva or food, as well as chest pain.

**Figure 5:**
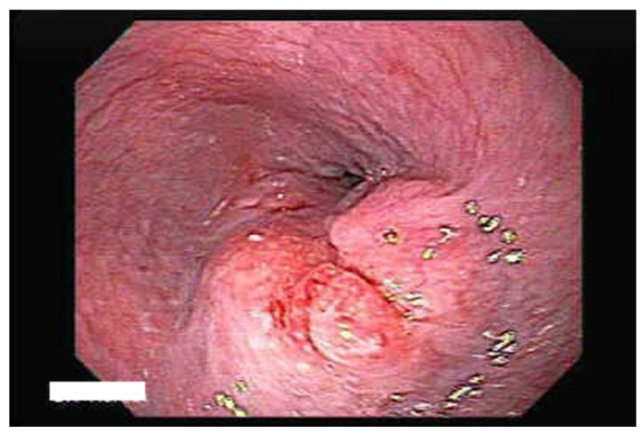
Esophageal adenocarcinoma.

Diagnosis: Endoscopic biopsy establishes the diagnosis of esophageal adenocarcinoma (Figure 5). Further staging is performed using cross-sectional imaging techniques, such as CT scans, endoscopic ultrasound, and FDG-PET scans [140].

Management: It depends on the stage of esophageal adenocarcinoma and the patient’s overall health. Early-stage cancer: A T1a lesion can be treated endoscopically by radiofrequency ablation. T1b lesion or higher needs to be treated by esophagectomy. Locally advanced cancer (T2 or higher, or node positive and non-metastatic) is treated by neoadjuvant chemotherapy (fluorouracil, leucovorin, oxaliplatin, and docetaxel) or chemoradiation followed by esophagectomy [141]. Metastatic, unresectable or recurrent cancer should be treated by chemotherapy plus immunotherapy (e.g., nivolumab or pembrolizumab) for HER2-negative, PD-L1 positive tumors, and trastuzumab-based regimens for HER2-positive tumors. Palliative radiotherapy or chemoradiotherapy may be used for symptom control in advanced cases [142]. Palliative esophageal fully covered self-expanding metal stent placement improves patients’ dysphagia and quality of life significantly in unresectable esophageal adenocarcinoma. It can also be used in patients receiving neoadjuvant therapy and waiting for definitive surgical treatment [143].

Prognosis: depends on the stage of the disease. Early-stage esophageal adenocarcinoma has an excellent prognosis. In case of locally advanced disease who receive perioperative chemotherapy plus surgery have a 3-year overall survival rate of 57.4% and a 5-year overall survival of 50.6%.

**Achalasia cardia or cardiospasm** is a rare, primary neurodegenerative esophageal smooth muscle spastic motor disorder characterized by hypertensive lower esophageal sphincter (LES), insufficient relaxation of the LES on wet swallows, and absent, aberrant, or disorganized peristalsis in the esophageal body. The incidence is approximately 1 in 100,000 people, and the prevalence is 10 per 100,000 people [144]. Males and females are equally affected. The disease can affect patients at any age, but most of the patients are diagnosed between the age of 25 to 60 years [145].

Etiology: The exact etiology of achalasia is unknown. Current evidence suggests that it is a multifactorial process causing autoimmune ganglionitis, possibly triggered by viral (varicella zoster, herpes simplex) infection in a genetically susceptible individual [146]. Genetic susceptibility is suggested by family clustering (i.e., the occurrence in monozygotic twins, siblings, and other first-degree relatives), as well as the presence of circulating antibodies against the myenteric plexus, and an immune cell infiltrate identified in the affected tissue, which suggests autoimmunity [147].

Pathogenesis: There is selective loss of inhibitory neurons, which produce nitric oxide and vasoactive intestinal peptide in the myenteric plexus of the distal esophagus and LES. As a result, unopposed excitatory activity leads to spastic LES with inadequate relaxation and aperistalsis of the esophagus [148]. Neuronal degeneration progresses in correlation with the different subtypes of achalasia, as shown on high-resolution manometry.

Symptoms: Progressive dysphagia to both solid and liquid diet is the most common (90% of cases) symptom of achalasia. Other symptoms with prevalence include regurgitation of undigested food in 75%, weight loss in 60% and heartburn in 40% [149]. The Eckardt Symptom Score (ESS) is a four-item self-reported achalasia symptom scoring tool used as a gold standard to grade symptom severity and response to treatment in both clinical and research studies, as shown in Table 1 [150]. Each item is graded on a scale of 0 to 3, with a total score ranging from 0 to 12. Following treatment of achalasia, a total score of 3 or less is considered treatment success, whereas a total score of more than 3 indicates treatment failure.

**Figure 6:**
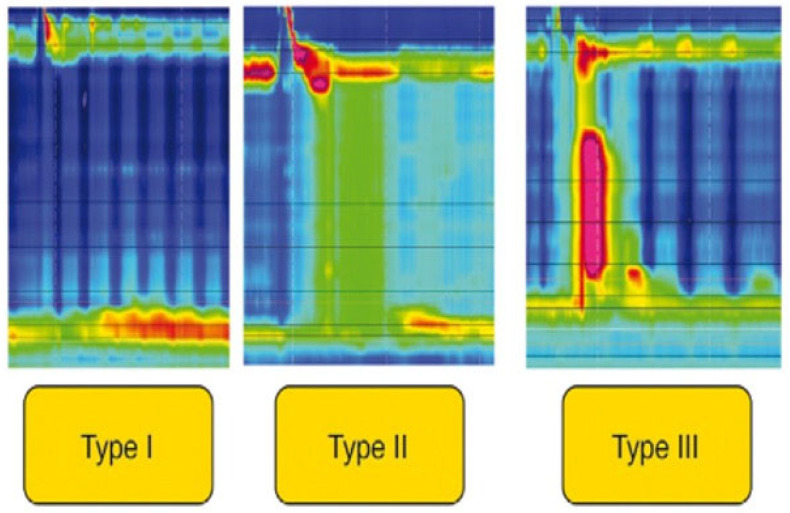
HRM classification of types of achalasia.

Diagnosis: Upper endoscopy is essential in suspected cases of achalasia to evaluate for any stricture at the esophagogastric junction and to rule out pseudoachalasia. It may show a dilated esophagus with retained food and fluid, as well as a spastic esophagogastric junction. However, in the early stages of achalasia, endoscopy may not reveal a dilated esophagus. Barium esophagogram may show a dilated esophagus with a bird’s beak appearance of the esophagogastric junction (EJG). However, in the early stage, a barium esophagogram may not reveal any positive findings. A timed barium esophagram (TBE) is preferable, as it quantifies esophageal emptying and can reveal abnormalities in early achalasia [151]. High-resolution manometry (HRM) is considered the gold standard for diagnosing and classifying achalasia. According to Chicago Classification Version 4.0 (CCv4.0), type I achalasia is characterized by elevated median integrated relaxation pressure (IRP) and 100% failed peristalsis. In type II achalasia, there is elevated median IRP, 100% failed peristalsis, and ≥20% swallows with panesophageal pressurization. Type III achalasia is diagnosed when there is elevated median IRP, ≥20% swallows with premature/spastic contraction, and no evidence of peristalsis (Figure 6) [152]. The Endoluminal Functional Lumen Imaging (EndoFLIP) impedance planimetry system can demonstrate the EJG distensibility index (DI) by simultaneously measuring the pressure and diameter of the EJG. An EGJ-DI ≥ 2.0 mm^2^/mmHg at 50–60 mL balloon distension with an EGJ diameter > 12 mm is considered normal for the LES as measured by FLIP. When HRM is inconclusive in diagnosing achalasia, particularly if the IRP is inconclusive, FLIP should be considered for further evaluation [153].

Management: It is mainly focused on relieving functional obstruction of the LES. It should be individualized depending on the subtypes of achalasia and the patient’s comorbidities. The different modalities include peroral endoscopic myotomy (POEM), laparoscopic Heller myotomy, pneumatic dilation, and botulinum toxin injection. In POEM, a submucosal tunnel is created in the esophagus using an endoscope. Selective myotomy of the inner circular muscle layer of the distal esophagus and LES is performed while preserving the outer longitudinal muscle layer. It is an effective and durable treatment of all types of achalasia, particularly type III achalasia (given the potential of extending the myotomy to the distal spastic segment of the esophagus), with a clinical success rate exceeding 90%. But the risk of acid reflux following POEM for achalasia is approximately 40–65% by objective criteria, and routine post-procedure acid suppression and surveillance are recommended [154]. Laparoscopic Heller myotomy is a minimally invasive surgery in which the outer longitudinal muscle layer of the LES, distal esophagus, and proximal stomach is cut, typically extending 6 cm proximally on the esophagus and 2–3 cm distally onto the gastric cardia. It is routinely combined with a partial fundoplication (such as Dor or Toupet). Heller myotomy is highly effective, especially for type I and type II achalasia, with a clinical success rate exceeding 85–90% but acid reflux can occur in 20% of cases without fundoplication [155]. In pneumatic dilation, graded endoscopic dilation of the LES is performed with a pneumatic balloon (balloon size 30, 35, or 40 mm) under fluoroscopic guidance. Usually, a 30 mm balloon is used first, and if there is no satisfactory response, a 35 or 40 mm balloon is used. Pneumatic dilation disrupts LES fibers. It is highly effective for type I and type II achalasia with success rates of 50 to 85% [156]. The risk of perforation following pneumatic dilation depends on the endoscopist’s experience, patient selection, and the size of the balloon used. In experienced hands, the perforation rate varies from 1.5% to 2.8% [157]. Endoscopic botulinum toxin injection into the LES is a non-invasive modality of treatment for achalasia. Botulinum toxin inhibits the release of acetylcholine and can lower the LES pressure, achieving a clinical success rate (Eckardt score ≤3) of 77% over 1 to 6 months. It is a safe procedure, but the effect is temporary, and it can cause inflammation and fibrosis of the LES, as well as rarely chest pain and mediastinitis. It is generally considered for patients who are poor candidates for POEM, Heller myotomy, or pneumatic dilation [158].

## 18. Schatzki’s Ring

This is a thin, circumferential, diaphragm-like mucosal structure located at the squamocolumnar junction of the distal esophagus, typically at or near the gastroesophageal junction. It is most associated with intermittent dysphagia to solid foods and is frequently found in conjunction with a sliding hiatal hernia [159]. The ring is composed of mucosa and submucosa, and is sharply demarcated from the surrounding esophageal tissue, distinguishing it from peptic strictures, which have a more gradual transition and are often associated with chronic gastroesophageal reflux disease (GERD).

Diagnosis is usually made by barium esophagram, which is more sensitive than endoscopy, as the ring may be obscured during endoscopic insufflation. The ring typically marks the proximal margin of a hiatal hernia.

Symptoms: Patients usually present with intermittent solid food dysphagia and, less commonly, acute food impaction.

The etiology is not fully understood but is thought to be multifactorial, with associations to GERD, hiatal hernia, and, in some cases, eosinophilic esophagitis.

Management: It is generally endoscopic dilation, which provides symptomatic relief in most patients; however, recurrence is common, and repeat dilations may be necessary. Acid suppression therapy with PPI should also be added [160].

## 19. Epiphrenic Diverticulum

This is a rare, pulsion-type diverticulum seen in the distal 10 cm of the esophagus just above the LES and the diaphragm. It is a pseudodiverticulum as its walls contain only mucosa and submucosa.

Pathogenesis: It is almost always associated with an underlying motility disorder, such as achalasia cardia or diffuse esophageal spasm. Increased intraluminal pressure leads to herniation of mucosa and submucosa through the muscular layer at a weak point in the esophageal wall and subsequent formation of an outpouching.

Symptoms: Patients remain asymptomatic majority of the time. Large diverticulum may cause regurgitation, aspiration, chest pain and dysphagia. But most of the symptoms are generally caused by an underlying motility disorder.

Diagnosis: Upper endoscopy and barium swallow can identify epiphrenic diverticulum. HRM should also be conducted to determine the underlying motility disorder [161].

Management: No treatment is indicated if the patient remains asymptomatic. In symptomatic cases, the underlying motility disorder should be managed, and the diverticulum should be treated by surgery. The surgical approach depends on the size and location of the diverticulum. The surgical modalities include diverticulectomy or laparoscopic transabdominal esophago-cardio-myotomy and fundoplication [162].

## 20. Esophageal Epidermoid Metaplasia (EEM), Also Known as Esophageal Leukoplakia

This is a rare condition that typically occurs in the distal or middle third of the esophagus. The exact etiology is unknown. The primary clinical concern is that it is a premalignant condition that can progress to squamous cell dysplasia and ultimately squamous cell carcinoma. Risk factors for developing EEM include cigarette smoking and alcohol intake.

Symptoms: Patient may remain asymptomatic or present with GERD or dysphagia.

**Figure 7:**
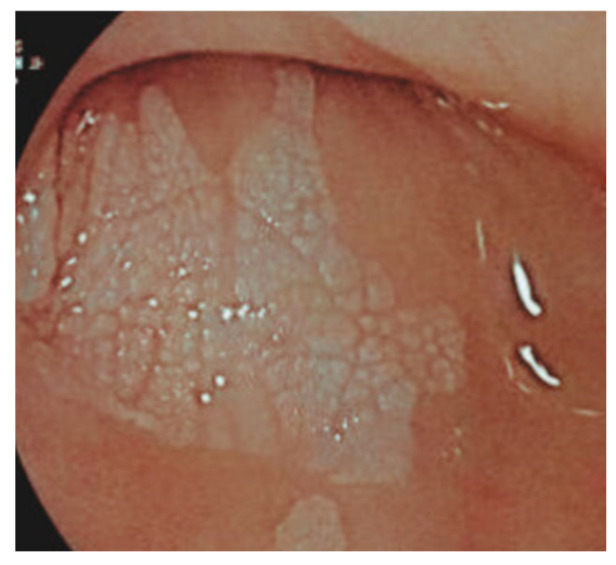
Esophageal epidermoid metaplasia.

Diagnosis: Upper endoscopy shows a white, scaly, and slightly nodular lesion (Figure 7) [163]. Biopsy of the lesion confirms the diagnosis. Histological findings include hyperkeratosis of the squamous epithelium and a prominent granular cell layer (orthokeratotic dysplasia) resembling epidermis of the skin [164].

Management: There are no formal treatment guidelines. As EEM is a premalignant condition, it is recommended to treat EEM by radiofrequency ablation, EMR, or ESD, particularly in the presence of dysplasia. Closed-endoscopic surveillance can be performed in non-dysplastic cases.

## 21. Esophageal Scleroderma

Up to 90% of patients with systemic sclerosis can develop gastrointestinal manifestations, and the esophagus is the most affected organ [165]. Esophageal scleroderma can occur with or without skin involvement (sine scleroderma).

Pathogenesis: Autoimmune injury to the blood vessels leads to the release of proinflammatory cytokines, including interleukin (IL) -2, IL-6, IL-9, IL-1β, and IL-17. Myofibroblasts also secrete transforming growth factor (TGF)-β, connective tissue growth factor, plasminogen activator inhibitor-1, and fibronectin 1. The inflammatory cascade and repair process lead to interstitial inflammation, fibrosis, and atrophy of the esophageal smooth muscles. As a result, esophageal dysmotility characterized by esophageal aperistalsis and decreased LES pressure occurs [166]. Long-standing esophageal dysmotility can cause GERD, esophageal stricture, Barrett’s esophagus, and, in rare cases, esophageal adenocarcinoma.

Symptoms: Heartburn due to GERD and dysphagia due to esophageal dysmotility are the main symptoms of esophageal scleroderma.

Diagnosis: Upper endoscopy may show evidence of erosive esophagitis. The diagnosis is confirmed by HRM, which shows absent contractility with an IRP of less than 15 mmHg. 24 h pH monitoring with impedance shows evidence of gastroesophageal acid reflux [167].

Management: It is mainly focused on the management of GERD with lifestyle modification and PPI or Potassium-competitive acid blocker (P-CAB) therapy. Nutritional support is essential as malnutrition is associated with increased mortality in this group of patients. A dietary consultation should be conducted, and oral, enteral, and parenteral support should be provided. Surgical intervention, such as fundoplication, is not recommended for patients with esophageal scleroderma [168].

## 22. Summary

Certain diseases are more likely to occur in specific parts of the esophagus. This predilection happens due to multiple factors, including anatomical, physiological, neurovascular supply, immunological, developmental, environmental, genetic, and other unknown factors. Patients are asymptomatic in the early stage of the disease process but become symptomatic as the disease becomes advanced. Imaging studies, endoscopy, and manometry with pH studies are the primary investigations used to diagnose various esophageal diseases. Treatment is targeted to control symptoms and resolve/stabilize the underlying pathogenesis. The prognosis varies depending on whether the disease is benign or malignant. A clear understanding of the more likely cause of the disease in specific locations of the esophagus, as seen during endoscopic procedures or imaging studies, helps gastroenterologists narrow down the differential diagnoses and better manage patients.

## Figures and Tables

**Table 1:** Eckardt score.

Scheme	Dysphagia	Regurgitation	Retrosternal Pain	Weight Loss (kg)
0	None	None	None	None
1	Occasional	Occasional	Occasional	<5
2	Daily	Daily	Daily	5–10
3	Each meal	Each meal	Each meal	>10

## Data Availability

Data are contained within the article.
